# Challenges in the classification and management of Asian youth-onset diabetes mellitus- lessons learned from a single centre study

**DOI:** 10.1371/journal.pone.0211210

**Published:** 2019-01-25

**Authors:** Toh Peng Yeow, Evelyn Su-Yin Aun, Chee Peng Hor, Shueh Lin Lim, Chong Hui Khaw, Nor Azizah Aziz

**Affiliations:** 1 Department of Medicine, Penang Medical College, Penang, Malaysia; 2 Department of Medicine, Division of Endocrinology, Penang General Hospital, Penang, Malaysia; 3 Clinical Research Centre, Seberang Jaya Hospital, Penang, Malaysia; 4 Department of Medicine, Kepala Batas Hospital, Penang, Malaysia; Temple University, UNITED STATES

## Abstract

It remains widely perceived that early-onset Type 2 Diabetes (T2D) in children and adolescents is rare and clinically distinct from Type 1 Diabetes (T1D). We studied the challenges of classifying subtypes of early-onset diabetes using clinical features and biomarkers, and management of these patients. We reviewed retrospectively the record of patients < 25 years old who attended the diabetes clinic in Penang General Hospital, Malaysia between 1^st^ December 2012 and 30^th^ June 2015. We examined their clinical features, C-peptide and pancreatic autoantibodies. Comparisons were made between T1D and T2D for magnitude, demographics, metabolic status and complications. We studied 176 patients with a mean age of 20 ± 3.7 years, 43.2% had T1D, 13.6% had T2D, and 13.6% had mixed features of both. When tested, pancreatic autoantibodies were positive in 59.4% of the T1D. T2D presented two years later than T1D at 14.3 years, 20% were asymptomatic at presentation, and 50% required insulin supplementation despite fasting c-peptide of > 250 pmol/L. HbA_1C_ of ≤ 8.0% (64 mmol/mol) was achieved in 30.3% of T1D, 58.3% of T2D on OAD and 16.7% of T2D on insulin. The T2D had greater cardiovascular risk with higher body mass index, more dyslipidaemia, higher blood pressure and earlier onset of nephropathy. The overlapping clinical features, variable autoimmunity, and beta-cell loss complicate classification of young diabetes. Pancreatic autoantibodies and C-peptide did not always predict diabetes subtypes nor respond to insulin. The poor metabolic control and high cardiovascular risk burden among the T2D highlight the need for population-based study and focused intervention.

## Introduction

Diabetes in childhood previously almost always equates Type 1 diabetes (T1D) but is increasingly recognized as a complex diagnosis. Early-onset type 2 diabetes mellitus (T2D) among children and adolescents is an emerging phenomenon worldwide [1, 2] with a higher burden in Asia than Europe and America combined [3]. T1D is due to beta-cell destruction leading to absolute insulin deficiency while T2DM is due to a progressive insulin secretory defect on the background of insulin resistance [4]. Prompt and accurate diagnosis allows clinicians to provide appropriate and effective treatment. However, distinguishing T1D from T2D is challenging for the clinicians due to their overlapping clinical features. The rising prevalence of childhood obesity affects all subtypes of diabetes while T2D can also present with severe hyperglycaemia and ketosis.

Malaysia has a high prevalence of T2D and multi-ethnic population comprising of predominantly Malay, Chinese and Indian ethnic groups. The population prevalence of diabetes was 17.5% in 2015 while the prevalence of diabetes among youth aged 18–19 years rose from 2.1% in 2011 up to 5.5% in 2015 [5, 6]. More than 90% of cases were detected only during screening, raising the possibility that they predominantly represent T2D. The Malaysian national registry on children and adolescents with diabetes reported 17.5% of them had T2D [7]. Despite this, the extent of the burden of T2D in the young remains understudied and often assumed to be low if compared to T1D.

We set out to examine the burden of early-onset diabetes and the challenges involved in classifying diabetes subtypes in a population like Malaysia with a high prevalence of diabetes. Comparisons are made between T1D and T2D for magnitude, demographics, metabolic status and complications. This understanding has immediate implications for the diagnosis and management of early-onset diabetes, as well as longer-term impact for public health.

## Materials and methods

### Ethics statement

The study was registered at Malaysian National Medical Research Register (NMRR) (reference number NMRR–13–927–17402). The study protocol was approved by the Medical Research and Ethics Committee (MREC), Ministry of Health Malaysia. As this study involved retrospective chart review with no direct patient contact, no written consent was obtained by the patients or guardians for their information to be stored in a database and used for research. MREC granted the waiver.

### Study design

We conducted a retrospective chart review from the 1^st^ December 2012 until the 30^th^ June 2015 at the Penang General Hospital (PGH). PGH is the largest public hospital in Penang. All patients with diabetes under the age of 25 years who attended the diabetes clinic in PGH were included. The charts were hand-searched, and information on gender, ethnicity, age at diagnosis, duration of diabetes, past medical history, family history of diabetes, mode of presentation and degree of hyperglycaemia at presentation were captured. Data on basic anthropometry, medication use, current glycaemic control, fasting lipid profile and presence of micro- and macro-vascular complications were captured from the most recent clinic review.

### Fasting C-peptide and pancreatic autoantibodies tests

Fasting C-peptide and pancreatic antibodies were routinely performed only for patients diagnosed in-house or for those with uncertain diagnosis. They were not routinely performed for patients who were referred in from elsewhere with an established diagnosis of T1D. The results were therefore not available in all patients. Serum C-peptide level was measured using the chemiluminescent method (Immulite, Siemen) at Pathology Department, Kuala Lumpur Hospital. We used fasting C-peptide of <250 pmol/l to define marked insulin deficiency [8]. The pancreatic antibodies were measured at the Allergy and Immunology Unit, Institute for Medical Research, Kuala Lumpur. These included anti-insulin (ELISA methods -Brand Biosystem), anti-glutamic acid decarboxylase (anti-GAD), anti-islet cell (anti-ICA) and anti-tyrosine phosphatase like-insulinoma antigen 2-antibodies (anti-IA-2) with ELISA methods- Medipan Medizym system. The cut-offs measurement to define seropositivity for these autoantibodies were as followed: anti-GAD (>10 IU/mL), anti-ICA (>1.0 binding index), anti-IA2 (>10 IU/mL) and anti-insulin (>10 U/mL).

### Classification of diabetes

Diabetes mellitus was diagnosed based on the WHO classification [9]. Patients were classified as T1D if they were clinically insulin dependent from diagnosis and as T2D if they were controlled on oral anti-diabetic (OAD) agent(s) alone for 3 or more years after diagnosis. Patients were also classified as T1D if they had fasting C-peptide of <250 pmol/L and as T2D if they had fasting C-peptide of ≥250 pmol/L at >3 years of diagnosis with negative pancreatic antibodies. Patients who failed to fulfil the aforementioned criteria for either T1D or T2D were classified as “uncertain”. Patients with diabetes secondary to another medical condition were classified as “secondary diabetes”.

### Cardiovascular risk markers

For patients aged 20 years or younger, body mass index (BMI) was categorized according to gender and age-percentile [10]. For patients aged more than 20 years, BMI was defined using standard set by the WHO [11]. Abnormal lipid profile was defined as total cholesterol, low-density lipoprotein (LDL-C) or triglyceride of more than 5.0 mmol/L, 3.0 mmol/L and 1.7 mmol/L respectively, or high-density lipoprotein (HDL-C) of less than 1.0 mmol/L [12]. Fasting remnant cholesterol was calculated by subtracting LDL-C and HDL-C from total cholesterol, and non-HDL cholesterol subtracted HDL-cholesterol from total cholesterol [12]. Metabolic syndrome was defined according to the WHO 1998 criteria [13].

Hypertension was diagnosed and classified based on age and gender. For those who aged 17 years or less, systolic blood pressure (SBP) and/ or diastolic blood pressure (DBP) ≥95^th^ centile(s) for age and gender was classified as hypertension; while SBP and/ or DBP between ≥90^th^ and <95^th^ centile(s) was classified as pre-hypertension [14]. For those who aged beyond 17 years, SBP ≥140 mmHg and/ or DBP ≥90 mmHg was classified as hypertension, while SBP ranging from 121 to 139 mmHg and/ or DBP from 81 to 89 mmHg was classified as pre-hypertension [15].

Albuminuria was defined as urine albumin-creatinine ratio of ≥2.5 mg/mmol for males and ≥3.5 mg/mmol for females.

### Statistical analyses

The data was collected and analysed using Statistical Package for the Social Sciences (SPSS) version 22.0. Comparative descriptive analyses were performed between T1D and T2D for demographics, presentation, family history, metabolic control and development of co-morbidities and complications. Chi-square tests were employed to investigate the relationship between types of diabetes (T1D and T2D) and patients’ demographic profiles, presenting symptoms, family history of diabetes, glycaemic control, BMI category, presence of hypertension, dyslipidemia, and complications of diabetes, as well as the use of anti-diabetic agents. Fischer exact tests were employed wherever assumptions for Chi-square tests were not fulfilled. Independent Student’s *t*-tests were employed to investigate the differences for age at diagnosis, duration of diabetes, blood pressure readings, glycaemic and metabolic parameters between T1D and T2D youths. Changes for glycaemic parameters for individuals were analysed using paired *t*-tests. All the data were de-identified before their use in analyses. All data are hosted in Penang General Hospital.

### Data availability

All data generated or analysed during this study are included in this article.

## Results

### Overview of study population

We registered 176 patients aged ≤25 years with diabetes mellitus. Male to female ratio was 1: 1.6 with predominantly Malay ethnicity (45%) followed by Chinese (39%) and Indian (16%). The mean age of diagnoses was 14±4.30 years old, with a mean disease duration of 6.2±4.26 years at the time of review. About 83% were treated with insulin (47% on insulin alone and 36% on the combination of insulin with OAD). The mean HbA_1c_ was 10.0±2.86% (86±31 mmol/mol) and only 27.3% managed to achieve HbA_1c_ of ≤8.0% (64 mmol/mol). Albuminuria and peripheral neuropathy were noted in 21% and 10.8%, respectively. Total cholesterol of >5.0 mmol/L, LDL-C of >3.0 mmol/L, triglyceride of > 5.0 mmol/L and HDL-C of <1.0 mmol/L occurred in 35.8%, 35.2%, 17.6% and 8.0% of youth with diabetes, respectively. Dyslipidemia, defined as having either one or a combination of these lipid abnormalities, was noted in 44.3% of all patients, of which 18.2% were on statin therapy. Upon applying age-adjusted definition, 5.7% had hypertension, 9.1% had isolated systolic hypertension while 32.4% had pre-hypertension. No patients had documented cardiovascular complications but 21% fulfilled definition for metabolic syndrome. Fasting C-peptide and pancreatic antibodies were tested in 48.9% and 58.5% of all patients, respectively. Of those tested, 51.1% had low C-peptide, and 58.5% were positive for at least 1 antibody.

### Classification of youth-onset diabetes

**Fig 1:**
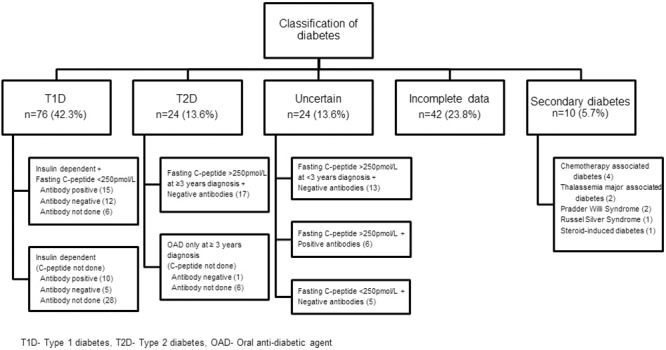
Classification of youth with diabetes.

Seventy-six (43.2%) and 24 (13.6%) patients fulfilled the study criteria for T1D and T2D, respectively (Fig 1). The characteristics, similarities, and differences between this 2 groups are discussed below.

Twenty-four (13.6%) patients were classified as “uncertain”. Of these, 13 had negative autoantibodies and normal C-peptide level but were not included as T2D as they were diagnosed for <3 years. However, 10 of these “probable T2D” already require insulin supplementation despite the short duration of diagnosis. Five other patients had negative antibodies but a low C-peptide level of <250 pmol/L. They were not insulin dependent at presentation (as in the case of classical T1D), but four of them subsequently required the addition of insulin to OAD therapy. Another six had positive anti-GAD, anti-ICA antibodies and normal C-peptide level and fulfilled definition for latent autoimmune diabetes of adults (LADA) [16].

Ten (5.7%) had diabetes secondary to genetic syndromes (3), thalassemia major (2), chemotherapy for malignancies (4), and steroid therapy for systemic lupus erythematosus (1). A further 42 patients were excluded from classifications as they did not have clear clinical features of either T1D or T2D and incomplete or absent C-peptide and autoantibodies results (incomplete data).

### Comparison between T1D and T2D

**Fig 2:**
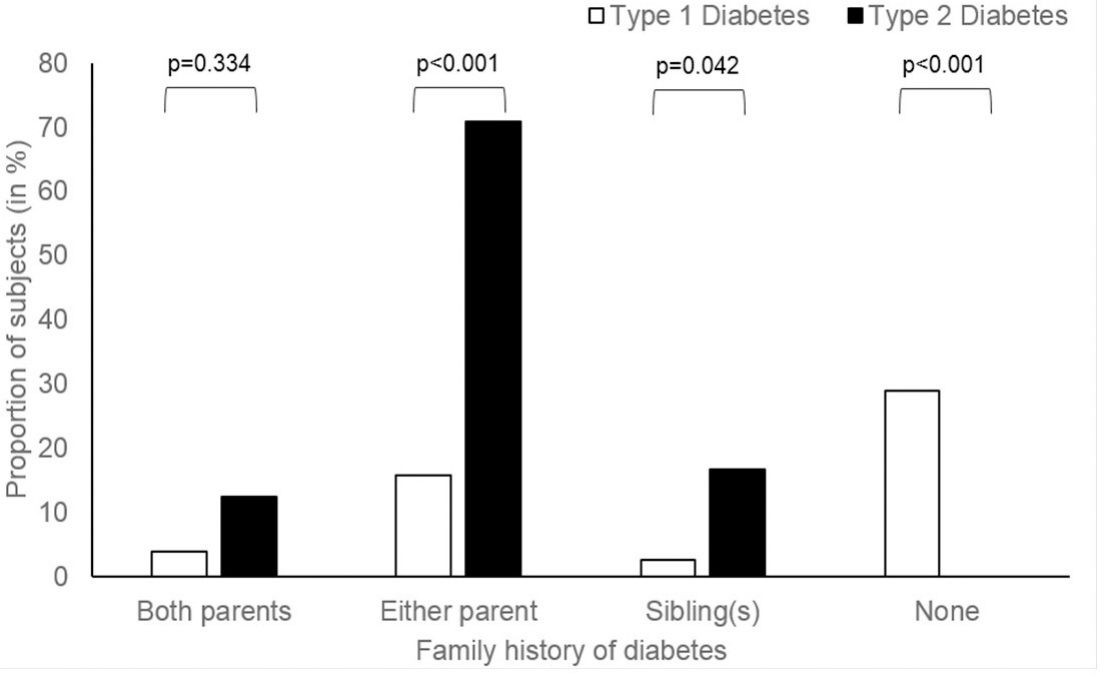
Family history of diabetes among youth with Type 1 and Type 2 diabetes.

Nearly 60% of the T1D and T2D patients were female (Table 1). Ethnic Chinese made up the majority of T1D, while ethnic Malay made up of the majority of T2D. T2D presented on average two years older than T1D at 14.3 years. One in five T2D were asymptomatic at presentation while all of the T1D had overt symptoms. Diabetic ketoacidosis (DKA) was the presenting diagnosis in 47% of T1D but none of the T2D. Acanthosis nigricans, a sign of insulin resistance, was present in nearly a third of T2D and 8% of T1D. All of the T2D have a family history of diabetes (in either first or second degree relatives), while 29% of T1D reported no family history of diabetes (Fig 2). Autoimmune disorders were more common among T1D with predominantly thyroid disorders and 1 autoimmune hepatitis. Both T1D and T2D had a similar prevalence of microvascular complications, with albuminuria being the most common (Table 2).

**Table 1:** Demographics, clinical characteristics and glycaemic controls among youth with T1D and T2D.

	T1D (n = 76); n (%)	T2D (n = 24); n(%)	p-value
**Gender**			
Male	32 (42.1)	10 (41.7)	0.970
Female	44 (57.9)	14 (58.3)	
**Ethnicity**			
Malay	24 (31.6)	12 (50.0)	0.151
Chinese	40 (52.6)	11 (45.8)	
Asian Indian	12 (15.8)	1 (4.2)	
**Age at time of review** (mean ± SD)	20.4±3.90	20.7±3.69	0.661
**Age at time of diagnosis** (mean ± SD)	12.3±4.79	14.3±3.52	0.061
**Duration of diabetes** (mean ± SD)	8.1±4.54	6.5±2.83	**0.042**
**Duration of follow up (years)** (mean ± SD)	4.7±2.67	4.2±2.3	**0.397**
**Presenting symptoms**			
Asymptomatic ^a^	0	5 (20.8)	**<0.001**
Diabetic ketoacidosis	36 (47.4)	0	**<0.001**
polyuria, polydispsia	32 (42.1)	10 (41.7)	0.108
Loss of weight	22 (28.9)	4 (16.7)	**0.048**
Blurring of vision ^a^	1 (1.3)	1 (4.2)	0.533
Infection ^a^	12 (15.8)	2 (8.3)	0.508
Lethargy	16 (21.1)	6 (25.0)	0.684
No records available	22 (28.9)	8 (33.3)	0.683
**Acanthosis nigricans**	6 (7.9)	8 (33.3)	**<0.001**
**Family history of diabetes** ^b^			
Both parents have DM ^a^	3 (3.9)	3 (12.5)	0.334
Either parent has DM	12 (15.8)	17 (70.8)	**<0.001**
Sibling(s) have DM ^a^	2 (2.6)	4 (16.7)	**0.042**
No family history of DM	22 (28.9)	0	**<0.001**
**Other autoimmune disorders** ^a^	11 (14.5)	1 (4.3)	0.284
**Glycaemic status at first clinic visit**			
Fasting glucose (mmol/L) (mean ± SD)	11.5±6.87	10.8±3.68	0.530
HbA_1c_ (%) (mmol/mol) (mean ± SD)	10.2±3.05 (88±33.3)	10.3±2.39 (89±26.1)	0.865
**Glycaemic control at most recent follow-up**			
Fasting blood glucose (mmol/L) (mean ± SD)	11.0±6.54	9.48±4.85	0.471
HbA_1c_ (%) (mmol/mol) (mean ± SD)	9.7±2.48 (83±27.1)	9.6±2.83 (81±30.9)	0.844
HbA_1c_ ≤ 6.5% (48 mmol/mol) ^a^	1 (1.3)	4 (16.7)	**0.013**
HbA_1c_ ≤ 8.0% (64 mmol/mol) ^a^	23 (30.3)	9 (37.5)	0.625
**Complications of Diabetes**			
Albuminuria ^a^	10 (13.2)	7 (29.2)	0.115
Peripheral neuropathy ^a^	9 (11.8)	3 (12.5)	1.000
Cataract, glaucoma or retinopathy ^a^	5 (6.6)	2 (8.3)	0.672
**Use of antidiabetic therapy** ^b^			
Insulin only	75 (98.7)	0	**<0.001**
OAD agents only ^a^	0	12 (50.0)	**<0.001**
Combination of insulin and OAD^a^	0	12 (50.0)	**<0.001**
Diet control only	0	0	-

^a^ Fischer exact test was employed for this variable.

^b^ Number in subgroup did not total up due to missing data.

**Table 2:** Cardio-metabolic risk markers at latest visit among youth with T1D vs T2D.

Risk markers	T1D (n = 76); n (%)	T2D (n = 24); n(%)	p-value
BMI (kg/m^2^) (mean ± SD)	22.9±3.50	30.3±7.93	**<0.001**
Underweight ^a^^**,**^^b^	2 (3.4)	1 (5.3)	1.000
Normal weight ^b^	44 (57.9)	5 (20.8)	**<0.001**
Overweight ^b^	10 (13.2)	3 (12.5)	0.949
Obese ^b^	2 (2.6)	10 (41.7)	**<0.001**
Total cholesterol (mmol/L) (mean ± SD)	5.1±1.24	5.2±1.40	0.789
LDL-C (mmol/L) (mean ± SD)	2.9±1.19	3.1±1.12	0.703
TG (mmol/L) (mean ± SD)	1.14±0.77	2.2±1.83	**0.025**
HDL-C (mmol/L) (mean ± SD)	1.6±0.55	1.3±0.35	**0.004**
TG:HDL-C ratio (mean ± SD)	0.8±0.69	2.0±1.80	**0.011**
Fasting remnant cholesterol (mmol/L) (mean ± SD)	0.5±0.43	0.8±0.60	**0.039**
Presence of dyslipidemia	24 (31.6)	16 (66.7)	**0.009**
Systolic blood pressure (mean ± SD)	117.6±17.59	129.7±13.33	**0.003**
Diastolic blood pressure (mean ± SD)	71.0±9.87	78.1±8.74	**0.003**
Normotension ^a^ ^b^	42 (55.3)	6 (25.0)	**0.009**
Pre-hypertension ^a^	23 (30.3)	11 (45.8)	0.213
Isolated systolic hypertension ^a^	6 (7.9)	3 (12.5)	0.683
Hypertension ^a^	1 (1.3)	3 (12.5)	**0.043**
Microalbuminuria (positive)	10 (13.2)	7 (29.2)	0.115
Metabolic syndrome	4 (5.3)	10 (41.7)	**<0.001**

LDL-C = low density lipoprotein cholesterol; HDL-C = high density lipoprotein cholesterol; TG = triglyceride

^a^ Fischer exact test was employed for this variable.

^b^ Number in subgroup did not total up due to missing data.

**Fig 3:**
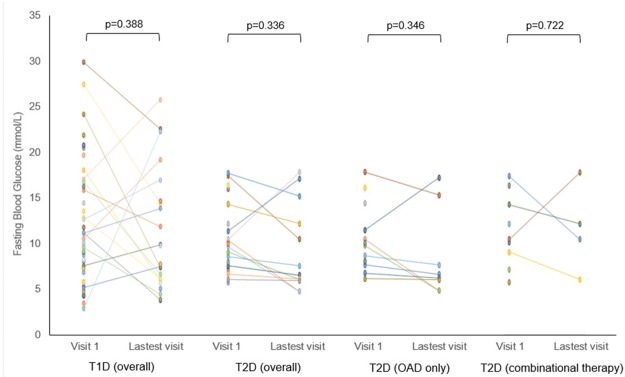
Serum fasting glucose among youth with diabetes at their initial and latest visits.

**Fig 4:**
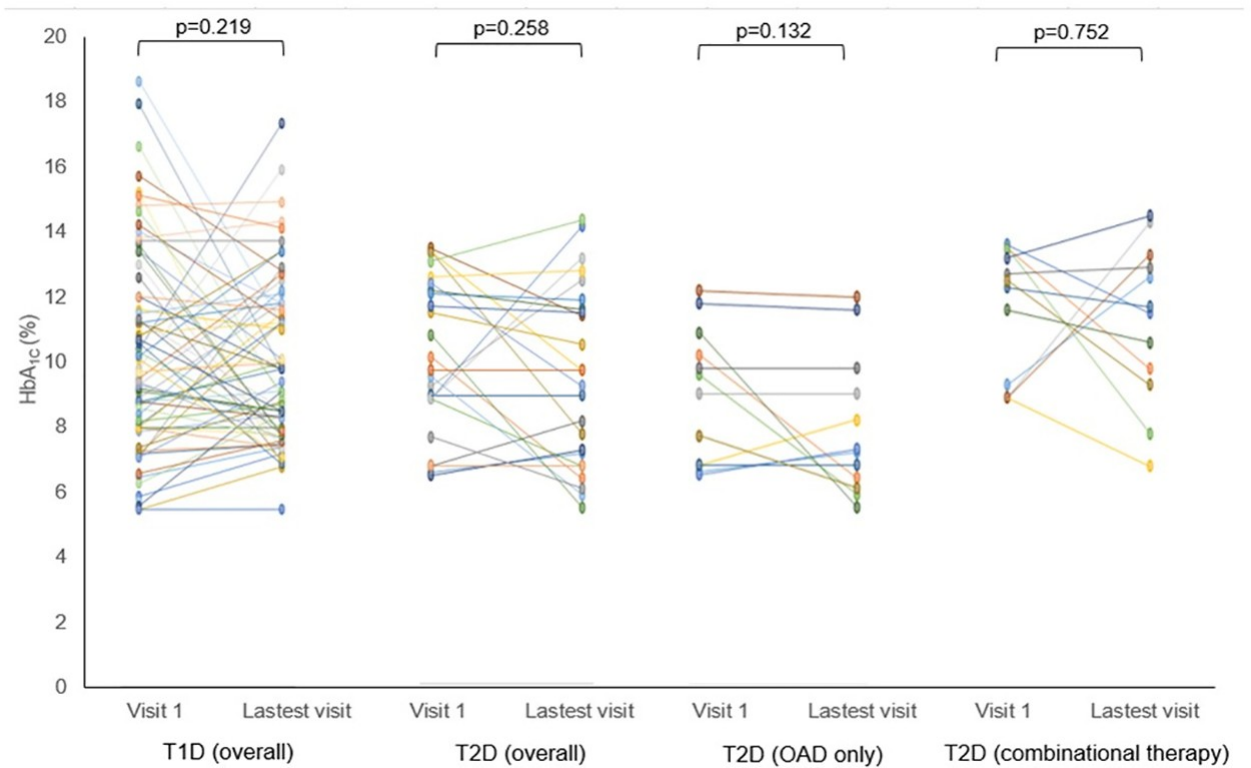
HbA_1c_ among youth with diabetes at their initial and latest visits.

The glycaemic control was equally poor between T1D and T2D at the first clinic visit as well as after a mean follow up of 4.9±2.80 years. Some T2D had more resistant hyperglycaemia than others. Insulin supplementation was needed in 50% of the T2D without resulting in an improvement in the fasting glucose (Fig 3) and HbA_1c_ (Fig 4). Only 16.7% of T2D supplemented with insulin achieved HbA_1C_ of ≤8.0% (64 mmol/mol), as compared to 58.3% of those treated with OAD alone. Subgroup analysis comparing T2D on OAD alone with T2D on insulin showed that both groups were similar in term of symptoms at presentation, demographic (gender, the age of diagnosis and duration of diabetes), family history, body mass index (BMI), lipid and blood pressure profiles. The only difference was the HbA_1c_ was higher among the T2D on insulin as compared to those on OAD alone, at initial clinic visit (11.1% (98 mmol/mol) vs 9.0% (75 mmol/mol), p = 0.005) and latest clinic visit (11.3% (100 mmol/mol) vs 8.0% (64 mmol/mol), p = 0.002).

Comparison was made between patients who achieved a Hba1c of ≤8.0% (64 mmol/mol) (“responders”) and those with HbA_1C_ of >8.0% (64 mmol/mol) (“non-responders”). Even with these loose definitions, only about one-third of T1D (n = 23) and T2D (n = 9) qualified as responders. The responders and non-responders in both T1D and T2D were similar in term of symptoms at presentation, gender, ethnicity, duration of diabetes and follow up, family history, BMI and blood pressure. The T1D responders were older than the non-responders (21.9±4.30 vs. 19.7±3.60 years, p = 0.03). The T2D non-responders had higher fasting glucose (8.1±1.42 vs. 12.6±3.62 mmol/L, p = 0.001) and HbA_1c_ (9.0±2.35% vs. 11.1±2.10%, p = 0.033) during the first visit. There were more insulin usage in the T2D non-responders group though it did not reach statistical significance. Despite the use of insulin, their HbA_1c_ remain above 8% (64 mmol/mol) at the latest visit. The T2D non-responders also had higher triglyceride level (1.3±0.82 vs. 2.9±2.13 mmol/L, p = 0.037) and more had microalbuminuria (0 vs. 46.7%, p = 0.022) than the responders.

Pancreatic antibodies profile were performed in 42 (of 76) T1D, 59.4% were seropositive for at least one of them, while 28.6% were positive for two autoantibodies and 14.3% for three autoantibodies. Anti-ICA was most commonly detected (52.9%), followed by anti-GAD (47.6%), anti-tyrosine phosphatase like-insulinoma antigen 2-antibodies (anti-IA2) (27.8%) and anti-insulin (2.9%) antibodies. About 40.5% (17 out of 42) of our T1D were seronegative for all autoantibodies.

The C-peptide level did not correlate with the need for insulin therapy. Fifty percent of the T2D required insulin supplementation for glycaemic control despite having fasting C-peptide of >250 pmol/L. Their daily insulin requirement ranged between 0.15 and 1.06 IU/kg.

### Cardiovascular risk markers

**Fig 5:**
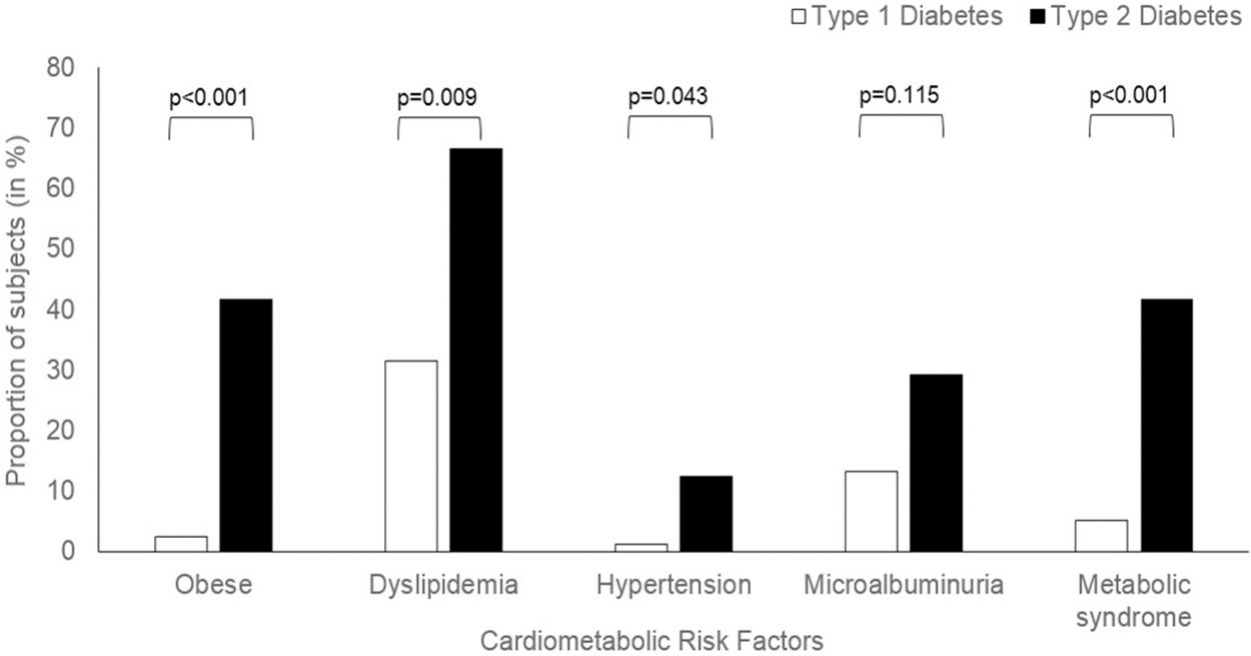
Cardio-metabolic risk among youth with Type 1 and Type 2 diabetes.

Youths with T2D were more likely to be obese (χ^2^ = 32.3, p<0.001) (Fig 5) and had higher BMI than T1D (30.3±7.93 versus 22.9±3.50 kg/m^2^, p<0.001) (Table 2). However, 20% of T2D youths had normal BMI, and 16% of T1D were overweight or obese.

Dyslipidaemia occurred in 31.6% of T1D youths and 66.7% of T2D youths (χ^2^ = 9.52, p = 0.009). Statin therapy was prescribed for 13.2% of T1D and 16.7% of T2D. T2D had significantly higher triglyceride, lower HDL-C, higher triglyceride to HDL-C ratio and fasting remnant cholesterol.

Blood pressure reading was captured from the most recent clinic visits with 10.4% of T1D and 9.1% of T2D receiving antihypertensive therapy. Systolic and diastolic blood pressure readings were significantly higher in T2D than T1D youth. Hypertension or isolated systolic hypertension occurred in 9.2% vs. 25.0% of T1D and T2D respectively (χ^2^ = 4.021, p = 0.076). Elevated albuminuria, also a sign of early nephropathy and increased cardiovascular risk, was noted in 13.2% of T1D and 29.2% of T2D, despite a shorter duration of disease in the T2D.

## Discussion

This study highlights the high disease burden among adolescents and youth with diabetes. Many of them have poorly controlled hyperglycaemia despite therapy with OADs and insulin and carry excess cardiovascular risks.

There appears to be a female preponderance, though not statistically significant, in both our T1D and T2D group. This is contrary to studies that showed male preponderance for Caucasians T1D, male preponderance for diabetes before the age of puberty and female preponderance only after menopause [17]. Women polycystic ovarian syndrome (PCOS) may exhibit pancreatic beta-cell dysfunction but our retrospective data did not contain reliable information about the Tanner staging and PCOS status of the subjects.

This study also highlights the challenges in differentiating T1D from T2D in early-onset diabetes in the youth due to significant overlap in clinical presentation, disease progression and biochemistry. However, selected clinical features at presentation could offer helpful clues. There were relatively more Malay in the T2D group and more Chinese in the T1D group. Patients who were asymptomatic at presentation were more likely to have T2D (about 20% of T2D were asymptomatic at presentation while all of the T1D had either polyuria, weight loss or ketosis). The average glucose and HbA_1c_ at presentation were as high in T2D as it was in T1D. Having a family history of diabetes especially in either parent greatly increased the odds of T2D. On the other hand, acanthosis nigricans, a sign of insulin resistance, was seen in the T2D group only.

C-peptide and pancreatic autoantibodies are not readily available (or affordable) in centres with a limited resource as in our setting. The results, even when available may not help to classify T1D from T2D accurately. After excluding subjects with secondary diabetes and those with incomplete data, about 19% (24/124 subjects) still could not be classified clearly into either T1D or T2D based on the clinical course, C-peptide level and pancreatic autoantibodies. This ambiguity in diagnosis may have contributed to delay in initiating appropriate treatment and subsequent poor glycaemic control.

The absence of pancreatic autoantibodies does not rule out T1D in our population. While the majority of T1D in the Caucasian population is driven by autoimmune beta-cell destruction, the role of autoimmunity in T1D is lower among Asians with T1D [18, 19]. In another Malaysian study, about 32% of patients with near or complete beta-cell destruction were seronegative for all pancreatic autoantibodies [19]. Similarly, 17 out of 42 T1D youth (40.5%) who were clinically insulin dependent in our study tested negative for these antibodies. These patients fit the definition of idiopathic T1D, a form of T1D not associated with autoantibodies, for which insulin treatment is required for survival although this requirement may be episodic. Idiopathic T1D is observed more commonly in African or Asian ancestry [20]. On the other hand, we had 6 patients with positive antibodies but detectable C-peptide. The autoimmune beta-cell destruction was slow in these patients as five out of 6 patients had fasting C-peptide of >250 pmol/L despite more than three years after diagnosis. While these patients may have LADA, they may also represent a spectrum of “antibody-positive T2D”. In a study by Nazaimoon et al, 7.5% of Malaysian clinically diagnosed as T2D were found to have positive anti-GAD antibody [18].

Low level of C-peptide did not always correlate clinically with the need for or response to insulin therapy in our patients. The serum C-peptide level reflects insulin secretion from pancreatic islet cells and residual insulin secretion function and has been suggested as a useful parameter in classifying diabetes. Katz et al. identified that a fasting C-peptide concentration **>**300 pmol/L had 83% sensitivity and 89% specificity for distinguishing paediatric T1D from T2D at diagnosis [21]. In another study of a Korean diabetes population, serum C-peptide level **<**200 pmol/L at diagnosis excludes T2D [22]. In this study, the fasting C-peptide of <250 pmol/l [8] did not always correlate clinically with the need for or response to insulin therapy. Five patients with C-peptide of <250 pmol/L and seronegative for pancreatic antibodies were not clinically insulin dependent (1 were on OAD only and four on OAD with insulin supplement). These patients could be either idiopathic T1D or “burn-out” T2D as the loss of beta-cell function is also known to be more rapid in youth-onset T2D compared to adult-onset T2D [2]. Neither does a “normal” level of C-peptide exclude the need for insulin therapy. Fifty percent of our T2D (all with fasting C-peptide of >250 pmol/L) needed to be prescribed insulin due to inadequate response to OAD therapy yet most did not show significant improvement.

This study also highlights the high burden of youth-onset T2D in our population. Applying the definition of T2D as persistent insulin production (by three years or more after diagnosis) and absence of autoimmune destruction, 13.6% of youths were categorized as T2D. The prevalence of T2D could be as high as 27.8% if the other patients who were managed as T2D were included (13 patients with negative beta-cell autoantibodies and normal C-peptide level but were at less than three years diagnosis and 12 patients who were treated with diet or OAD alone but were diagnosed for less than three years and did not have C-peptide or antibody testing).

The poor glycaemic control for many of the T1D and T2D even after treatment is of great concern. Only about a third of our patients achieved HbA_1c_ of <8% (64 mmol/mol) after about four years of follow-up. Intermittent compliance with pharmacotherapy and clinic visits may have contributed to the poor control observed, but we were unable to reliably capture the compliance information given the retrospective nature of our study. The older age observed in the T1D responders may be due to better compliance with therapy in the older patients. In the TODAY trial, 39% of the participants failed to reach HbA_1c_ of <8% (64 mmol/mol) despite treatment with metformin, rosiglitazone and intensive lifestyle coaching under the supportive trial environment [23, 24]. A worrying 62.5% of our T2D cohort had similar treatment failure, with a longer duration of disease, and many were treated with insulin therapy. However, there may be inherent differences within the broad group of T2D. A subset of our T2D appears to respond well to OAD alone with 58.3% achieved HbA_1C_ of ≤8.0% (64 mmol/mol), while the other had poor glycaemic control despite the intensification of therapy with insulin, only 16.7% achieved the same HbA_1C_ target. The T2D non-responders carry excess cardiovascular risk burden, as evidenced by higher triglyceride and microalbuminuria. Further studies are needed to understand if there were subtypes of T2D with different pathophysiology necessitating different treatment approach. Asian youths with T2D have preserved glucagon-like peptide-1 response to oral glucose, but decreased incretin effect, beta-cell function and insulin sensitivity [25]. Further studies are needed to elucidate the possibility of GLP-1 resistance in youth with T2D and role of incretin-based therapy.

Some of the T2D were diagnosed incidentally and denied any symptoms at diagnosis. The prevalence of diabetes among Malaysian youth aged 18–24 were 5.5–5.9% with 90% detected during screening [6]. The early identification of T2D is important not least because development and progression of clinical complications are more rapid, and associated with greater mortality when the onset of T2D is early [26]. Obesity alone may not be a sensitive risk marker as the increased risk of diabetes in the Asian youths appears to start at lower BMI. The mean BMI in our population of young T2D was 30.3 kg/m^2^ compared to above 32 kg/m^2^ as reported from the West [27, 28], and one-quarter of our young T2D have normal BMI or underweight. The lack of obesity however, was accompanied by a similarly high burden of co-morbidities and complications as those observed in other populations [29]. About 25% of our young T2D were hypertensive, and another 45.8% fell into the pre-hypertension category. The higher prevalence of hypertension in the young T2D compared to young T1D may be driving early onset of nephropathy. Despite a shorter duration of diabetes and similar HbA_1c_, 29.2% of young T2D had albuminuria, compared to 13.2% of T1D. This high rate of nephropathy is in keeping with observation in other studies [30, 31]. Youth with T2D were found to have a 23-fold and 4-fold increased risk of renal failure compared to healthy control and youth with T1D [32]. Our T2D patients also had adverse lipid profile with low HDL-C, high triglyceride, triglyceride to HDL-C ratio and fasting remnant cholesterol, which are significant for coronary artery diseases [33, 34]. The high burden of dyslipidaemia in our study population is similar to other studies [35, 36]. These early presences of hypertension, albuminuria and dyslipidaemia may translate into excessive future cardiovascular risks. Adults with early-onset T2D have 14-fold higher risk of developing myocardial infarction than the control subjects [37]. Despite similar glycemic exposure and shorter disease duration, youth-onset T2D was associated with more complications and twice the mortality rate when compared with T1D [35].

Several limitations of the study have to be mentioned. We restricted the definitions of T1D and T2D. While this resulted in a smaller number of subjects in each group, it enabled greater certainty in the comparative analyses. The prevalence of obesity and hypertension in this young population are also made more accurate by applying age- and gender-specific cut-off in the definition. The study was undertaken at a referral centre which would have imposed referral biases. The retrospective chart review nature of our data may not reliably represent the target population. Serum C-peptide level and antibodies were not performed for all subjects, and we did not rule out Maturity Onset Diabetes of the Youth (MODY). However, our study highlights that the C-peptide and antibodies results (when available) do not always accurately predict clinical progression and response to therapy. Genetic testing for MODY is not readily available in this region and prohibitively expensive. A clinical prediction model has been developed (MODY probability calculator) to guide clinicians in the diagnosis of young people with diabetes, but its validity is yet untested in the Malaysian population [38].

## Conclusions

This study highlights the high disease burden among adolescents and youth with diabetes. With every three patients diagnosed with T1D in this study, there were at least one patient with T2D and one more who could not be neatly classified. Patients who were asymptomatic at presentation, with a family history of T2D among the first degree relative and had acanthosis nigricans were more likely to have T2D. Pancreatic autoantibodies and C-peptide level on the other hand, failed to reliably predict needs for or response to insulin therapy in many patients. The T2D appear to bear a higher disease burden compared to the T1D, with more dyslipidaemia, hypertension, metabolic syndrome and microalbuminuria, despite a shorter duration of diabetes and similarly high HbA_1c_. Poorly controlled diabetes in adolescents and youth poses a serious public-health challenge for the coming decades. There is a need for more comprehensive population-based estimates of diabetes incidence among Malaysian youth. There is also a need for further study to understand the pathophysiology and factors driving this early manifestation of disease and complications.

## References

[1] D’Adamo E, Caprio S. Type 2 diabetes in youth: Epidemiology and pathophysiology. Diabetes Care. 2011;34(Supplement 2):S161–S5. 10.2337/dc11-s212 21525449 PMC3632155

[2] Nadeau KJ, Anderson BJ, Berg EG, Chiang JL, Chou H, Copeland KC, et al Youth-Onset Type 2 Diabetes Consensus Report: Current Status, Challenges, and Priorities. Diabetes Care. 2016;39(9):1635–42. 10.2337/dc16-1066 27486237 PMC5314694

[3] Chan JC, Malik V, Jia W. Diabetes in Asia: epidemiology, risk factors, and pathophysiology. JAMA. 2009;301 10.1001/jama.2009.726 19470990

[4] Standards of Medical Care in Diabetes– 2015. Diabetes Care. 2015;38.

[5] Mustapha FI. Current burden of diabetes in Malaysia (unpublished data). 15th National Institute of Health Scientific Meeting; Kuala Lumpur, Malaysia: Ministry of Health, Malaysia; 2012.

[6] Institute for Public Health (IPH). National Health and Morbidity Survey 2015 (NHMS 2015). Kuala Lumpur: Institute for Public Health, 2015.

[7] Fuziah MZ, Hong JY, Zanariah H, Harun F, Chan SP, Rokiah P, et al A national database on children and adolescent with diabetes (e-DiCARE): results from April 2006 to June 2007. The Medical journal of Malaysia. 2008;63 Suppl C:37–40. Epub 2009/02/21. . 19230245

[8] Jones AG, Hattersley AT. The clinical utility of C-peptide measurement in the care of patients with diabetes. Diabetic medicine: a journal of the British Diabetic Association. 2013;30(7):803–17. Epub 2013/02/19. 10.1111/dme.12159 . 23413806 PMC3748788

[9] WHO. Definition and diagnosis of diabetes mellitus and intermediate hyperglycaemic: a report of a WHO/ IDF consultation. Geneva, Switzerland: 2006.

[10] CDC. About Child & Teen BMI Atlanta, United States: Division of Nutrition, Physical Activity, and Obesity, National Center for Chronic Disease Prevention and Health Promotion. http://www.cdc.gov/healthyweight/assessing/bmi/childrens_bmi/about_childrens_bmi.html.

[11] National Institute of Health. The Practical Guide: Identification, Evaluation, and Treatment of Overweight and Obesity in Adults. 2000.

[12] Nordestgaard BG, Langsted A, Mora S, Kolovou G, Baum H, Bruckert E, et al Fasting is not routinely required for determination of a lipid profile: clinical and laboratory implications including flagging at desirable concentration cut-points-a joint consensus statement from the European Atherosclerosis Society and European Federation of Clinical Chemistry and Laboratory Medicine. European heart journal. 2016;37(25):1944–58. Epub 2016/04/29. 10.1093/eurheartj/ehw152 . 27122601 PMC4929379

[13] Huang PL. A comprehensive definition for metabolic syndrome. Disease models & mechanisms. 2009;2(5–6):231–7. Epub 2009/05/02. 10.1242/dmm.001180 . 19407331 PMC2675814

[14] National High Blood Pressure Education Program Working Group on High Blood Pressure in Children and Adolescents. The Fourth Report on the Diagnosis, Evaluation, and Treatment of High Blood Pressure in Children and Adolescents. Pediatrics. 2004;114(Supplement 2):555–76. 15286277

[15] Chobanian AV, Bakris GL, Black HR, Cushman WC, Green LA, Izzo JL, et al Seventh Report of the Joint National Committee on Prevention, Detection, Evaluation, and Treatment of High Blood Pressure. Hypertension. 2003;42(6):1206–52. 10.1161/01.HYP.0000107251.49515.c2 14656957

[16] Stenstrom G, Gottsater A, Bakhtadze E, Berger B, Sundkvist G. Latent autoimmune diabetes in adults: definition, prevalence, beta-cell function, and treatment. Diabetes. 2005;54 Suppl 2:S68–72. Epub 2005/11/25. . 16306343 10.2337/diabetes.54.suppl_2.s68

[17] Mauvais-Jarvis F. Gender differences in glucose homeostasis and diabetes. Physiology & behavior. 2018;187:20–3. Epub 2017/08/28. 10.1016/j.physbeh.2017.08.016 . 28843891 PMC5826763

[18] Wan Nazaimoon WM, Faridah I, Singaraveloo M, Ismail IS, Wan Mohamad WB, Letchuman R, et al Prevalence of glutamic acid decarboxylase antibodies amongst young Malaysian diabetics. Diabetes Research and Clinical Practice. 1999;43(1):59–66. 10199589 10.1016/s0168-8227(98)00108-9

[19] Wan Nazaimoon WM, Azmi KN, Rasat R, Ismail IS, Singaraveloo M, Mohamad WB, et al Autoimmune markers in young Malaysian patients with type 1 diabetes mellitus. Med J Malaysia. 2000;55(3):318–23. 11200711

[20] Association AD. 14. Diabetes Care in the Hospital. Diabetes Care. 2017;40(Supplement 1):S120–S7. 10.2337/dc17-S017 27979901

[21] Katz LE, Jawad AF, Ganesh J, Abraham M, Murphy K, Lipman TH. Fasting c-peptide and insulin-like growth factor-binding protein-1 levels help to distinguish childhood type 1 and type 2 diabetes at diagnosis. Pediatr Diabetes. 2007;8(2):53–9. Epub 2007/04/24. 10.1111/j.1399-5448.2007.00236.x . 17448127

[22] Cho MJ, Kim MS, Kim CJ, Kim EY, Kim JD, Kim EY, et al Fasting serum C-peptide is useful for initial classification of diabetes mellitus in children and adolescents. Annals of Pediatric Endocrinology & Metabolism. 2014;19(2):80–5. 10.6065/apem.2014.19.2.80 . 25077090 PMC4114050

[23] Allen DB. TODAY—A stark glimpse of tomorrow. New Engl J Med. 2012;366(24):2315–6. 10.1056/NEJMe1204710 22540913

[24] TODAY Study Group. A clinical trial to maintain glycemic control in youth with Type 2 diabetes. New Engl J Med. 2012;366(24):2247–56. 10.1056/NEJMoa1109333 . 22540912 PMC3478667

[25] Yeow TP, Pacini G, Tura A, Hor CP, Lim SL, Tan FHS, et al Preserved glucagon-like peptide-1 responses to oral glucose, but reduced incretin effect, insulin secretion and sensitivity in young Asians with type 2 diabetes mellitus. BMJ Open Diabetes Research & Care. 2017;5(1). 10.1136/bmjdrc-2016-000352 28321312 PMC5353273

[26] Constantino MI, Molyneaux L, Limacher-Gisler F, Al-Saeed A, Luo C, Wu T, et al Long-term complications and mortality in young-onset diabetes: type 2 diabetes is more hazardous and lethal than type 1 diabetes. Diabetes Care. 2013;36(12):3863–9. Epub 2013/07/13. 10.2337/dc12-2455 mc3836093. 23846814 PMC3836093

[27] Gungor N, Bacha F, Saad R, Janosky J, Arslanian S. Youth Type 2 Diabetes: Insulin resistance, β-cell failure, or both? Diabetes Care. 2005;28(3):638–44. 10.2337/diacare.28.3.638 15735201 PMC3428068

[28] McQuaid S, O’Gorman DJ, Yousif O, Yeow TP, Rahman Y, Gasparro D, et al Early-onset insulin-resistant diabetes in obese Caucasians has features of typical Type 2 diabetes, but 3 decades earlier. Diabetes Care. 2005;28(5):1216–8. 10.2337/diacare.28.5.1216 15855595

[29] Pinhas-Hamiel O, Zeitler P. Acute and chronic complications of type 2 diabetes mellitus in children and adolescents. The Lancet. 2007;369(9575):1823–31. S0140-6736(07)60821-6. 10.1016/S0140-6736(07)60821-6 17531891

[30] Maahs DM, Snively BM, Bell RA, Dolan L, Hirsch I, Imperatore G, et al Higher prevalence of elevated albumin excretion in youth with Type 2 than Type 1 diabetes: The SEARCH for diabetes in youth study. Diabetes Care. 2007;30(10):2593–8. 10.2337/dc07-0450 17630264

[31] Yoo EG, Choi IK, Kim DH. Prevalence of microalbuminuria in young patients with type 1 and type 2 diabetes mellitus. J Pediatr Endocrinol Metab. 2004;17(10):1423–7. . 15526721 10.1515/jpem.2004.17.10.1423

[32] Dart AB, Sellers EA, Martens PJ, Rigatto C, Brownell MD, Dean HJ. High burden of kidney disease in youth-onset type 2 diabetes. Diabetes Care. 2012;35(6):1265–71. 10.2337/dc11-2312 22432116 PMC3357249

[33] Varbo A, Benn M, Smith GD, Timpson NJ, Tybjaerg-Hansen A, Nordestgaard BG. Remnant cholesterol, low-density lipoprotein cholesterol, and blood pressure as mediators from obesity to ischemic heart disease. Circulation research. 2015;116(4):665–73. Epub 2014/11/21. 10.1161/CIRCRESAHA.116.304846 . 25411050

[34] da Luz PL, Favarato D, Faria-Neto JR Jr., Lemos P, Chagas AC. High ratio of triglycerides to HDL-cholesterol predicts extensive coronary disease. Clinics (Sao Paulo, Brazil). 2008;63(4):427–32. Epub 2008/08/23. 10.1590/S1807-59322008000400003 . 18719750 PMC2664115

[35] Constantino MI, Molyneaux L, Limacher-Gisler F, Al-Saeed A, Luo C, Wu T, et al Long-term complications and mortality in young-onset diabetes: Type 2 diabetes is more hazardous and lethal than type 1 diabetes. Diabetes Care. 2013 10.2337/dc12-2455 23846814 PMC3836093

[36] Song SH. Complication characteristics between young-onset type 2 versus type 1 diabetes in a UK population. BMJ Open Diabetes Research & Care. 2015;3(1). 10.1136/bmjdrc-2014-000044 25713725 PMC4336407

[37] Hillier TA, Pedula KL. Complications in young adults with early-onset Type 2 diabetes: Losing the relative protection of youth. Diabetes Care. 2003;26(11):2999–3005. 10.2337/diacare.26.11.2999 14578230

[38] Amed S, Pozzilli P. Diagnosis of diabetes type in children and young people: challenges and recommendations. The Lancet Diabetes & Endocrinology. 4(5):385–6. 10.1016/S2213-8587(16)00080-2 27053420

